# Increased Neutrophil Extracellular Trap Formation in Uremia Is Associated with Chronic Inflammation and Prevalent Coronary Artery Disease

**DOI:** 10.1155/2017/8415179

**Published:** 2017-03-27

**Authors:** Jwa-Kyung Kim, Chang-won Hong, Mi Jin Park, Young Rim Song, Hyung Jik Kim, Sung Gyun Kim

**Affiliations:** ^1^Department of Internal Medicine and Kidney Research Institute, Hallym University Sacred Heart Hospital, Anyang, Republic of Korea; ^2^Department of Clinical Immunology, Hallym University Sacred Heart Hospital, Anyang, Republic of Korea; ^3^Department of Physiology, School of Medicine, Kyungpook National University, Daegu, Republic of Korea

## Abstract

*Background.* Neutrophils are involved in the pathogenesis of atherosclerosis by neutrophil extracellular trap (NET) formation. We hypothesized that the NET formation of neutrophils might be changed in end-stage renal disease (ESRD) patients, explaining their higher incidence of coronary artery diseases (CAD). *Method.* A cross-sectional study was performed in 60 maintenance hemodialysis (MHD) patients, 30 age- and sex-matched healthy individuals (HV, negative control), and 30 patients with acute infection (positive control). Neutrophil activation and function were measured with reactive oxygen species (ROS) activity, degranulation, NET formation, and phenotypical changes. *Result.* Compared with HV, neutrophils extracted from MHD patients displayed significantly increased levels of basal NET formation, ROS production, and degranulation, suggesting spontaneous activation in uremia. Also, an increase in citrullinated histone H3 was detected in this group compared to the HV. And neutrophils from HV were normal CD16^bright^/CD62L^bright^ cells; however, neutrophils from MHD were CD16^bright^/CD62L^dim^, similar to those from patients with acute infections. Interestingly, multivariate analyses identified the prevalent CAD and neutrophil counts as independent determinants of baseline NET formation (*β* = 0.323, *p* = 0.016 and *β* = 0.369, *p* = 0.006, resp.). *Conclusions.* Uremia-associated-increased NET formation may be a sign of increased burden of atherosclerosis.

## 1. Introduction

Patients with end-stage renal disease (ESRD) are clearly at a high risk of death, approximately ten times greater than those of the general population. Death in ESRD patients generally results from cardiovascular (CV) disease or infection, and increasing evidence suggests that immune dysfunction contributes to both complications [1, 2]. In fact, alterations in the immune system are well established in ESRD patients [2, 3]. The retention of uremic toxins and cytokines is regarded as a key contributor of immune dysfunction by inducing oxidative stress and inflammation [1–4], rendering the patients more susceptible to infections [5] and atherosclerosis [6–8].

Among the complex cascade of immune system, the importance of innate immunity, especially the role of neutrophils, has been recently emphasized. Neutrophils are the most abundant human immune cell that kills bacteria. However, recently, the potent role of neutrophils in chronic inflammation has been also emphasized [9]. Particularly in ESRD patients, the bactericidal abilities are known to be reduced despite the increased basal activation state of neutrophils (neutrophil priming), suggesting the dysfunction of neutrophils [10–13]. Rather, the excessive activation and increased apoptosis in uremic condition cause low-grade inflammation, endothelial dysfunction, and atherosclerosis [14]. The formation of neutrophil extracellular traps (NETs), a newly discovered novel cell death program, could support the putative roles of neutrophils [15, 16].

NETs are networks of extracellular DNA fibers, primarily composed of histones and antimicrobial proteins released from neutrophils [17, 18]. Initially, NETs were described as an antimicrobial mechanism whereby invading pathogens become entrapped in the web-like structure [18]. However, increasing evidence suggests that NETs play more significant roles in autoimmune or inflammatory pathologies by serving as endogenous stimuli (e.g., alarmins) [19]. NETs induce sterile inflammation in multiple diseases, including vasculitis [20], rheumatoid arthritis [21], systemic lupus erythematosus [22, 23], coronary artery disease (CAD) [24], and atherosclerosis [15], which can be harmful to the host. Previous studies have demonstrated that during NET formation, histone proteins can be citrullinated and this histone hypercitrullination mediates chromatin decondensation and NET release [25]. In fact, citrullination of histone is an early response to inflammatory stimuli in neutrophils, and citrullinated histone H3 (Cit H3) has been identified as a component of NETs [25, 26].

Despite such studies focusing on inflammatory diseases, no studies have been performed in hemodialysis (HD) patient. HD patients are a specialized population characterized by chronic low-grade inflammation and increased oxidative stress with abundant uremic toxins [1, 4, 27]. Thus, we hypothesized that the NET formation might be changed in HD patients, leading to the higher prevalence of cardiovascular diseases in this population.

## 2. Materials and Methods

### 2.1. Study Population

In this cross-sectional study, we evaluated 60 maintenance HD (MHD) patients (35 men, 25 women) who had received HD three times per week for at least 3 months prior to the study. MHD patients with a history of active infection within 2 weeks, malignancy, decompensated liver cirrhosis, or those taking immune suppressants were excluded. All patients underwent regular HD for 3.5–4 h, three times per week, using standard bicarbonate dialysis (sodium 138 mmol/L, HCO_3_ 35–40 mmol/L, potassium 1.5 mmol/L, calcium 1.25 mmol/L, and magnesium 0.75 mmol/L) and semisynthetic membranes (dialysis filter surface areas 1.5–2.1 m^2^). Baseline demographic data, including age, sex, and smoking status, as well as clinical data regarding the underlying cause of renal disease, comorbidities (diabetes, hypertension, CAD, peripheral arterial disease, and cerebrovascular accident), and medication history, were obtained. In this study, the presence of CAD was restricted to the angiographically documented significant coronary stenosis with coronary intervention (percutaneous coronary intervention or coronary artery bypass graft surgery). Biochemical analyses of white blood cells (WBC), neutrophils, lymphocytes, and levels of hemoglobin, serum albumin, total cholesterol, urea nitrogen, creatinine, uric acid, high-sensitivity C-reactive protein (hs-CRP), and beta-2 microglobulin were measured.

In addition, 30 age- and sex-matched healthy individuals (HV, negative control), as well as 30 age- and sex-matched patients with acute infection (positive control), were also included in the study. The inclusion criteria for HV were individuals with normal physical and neurological examinations, without history of chronic comorbid conditions such as hypertension, diabetes, and hyperlipidemia. For positive controls, blood samples from patients with full-blown infection were acquired. Patients admitted to intensive care unit for severe sepsis or septic shock were included, and blood samples were acquired within 24 hours after admission. The study protocol was approved by the Institutional Review Board at Hallym University Sacred Heart Hospital, and informed consent was obtained from all patients.

### 2.2. Isolation of Neutrophils

Whole blood was collected following cannulation of the vascular access but before the initiation of dialysis. Peripheral blood samples were collected using a needle and syringe by gently aspirating into EDTA vacuum container tubes. To avoid in vitro activation or modification, blood samples were processed immediately following collection, without manipulation. Neutrophils were isolated as previously described using dextran sedimentation of red blood cell (RBC) pellets [28]. Cells were collected by centrifugation and found to contain >95% neutrophils. For patients with acute infections, samples were obtained within 24 h after hospital admission. To evaluate neutrophil function, reactive oxygen species (ROS) activity, degranulation, and NET formation were assessed at baseline and upon stimulation.

### 2.3. Flow Cytometric Analysis of Surface Receptors and Activation Markers in Blood Neutrophils

For analysis of phenotype, freshly isolated cells were incubated with the CD15 (clone HI98), CD16 (clone NKP15), and CD 62L (clone DREG-56) to find out surface expression of those markers. In addition, to find degranulation and activation status of neutrophils, the expression of various granule markers such as CD 35 (clone E11), CD63 (clone H5C6), and CD 66b (clone G10F5) antibodies (BD Biosciences, San Jose, CA, USA) was evaluated at 4°C for 30 min in the dark. Cells were then washed with PBS twice and resuspended in 0.5 mL of 0.5% paraformaldehyde (Sigma Chemical Co, Poole, UK). Flow cytometry was performed using a FACScalibur (BD Biosciences, San Jose, CA, USA) based on CD15-positive neutrophils. For all cases, at least 15,000 cells were acquired for each case and analyzed by gating on neutrophils.

### 2.4. NET and ROS Quantification Assay

The formation of NETs was evaluated at basal state and at activated state using the membrane impermeable DNA-binding dye, SYTOX green (Molecular Probes, Invitrogen Life Technologies). Experiments were performed in 96-well culture plates. Freshly isolated neutrophils (2 × 10^5^/well) were stimulated with a protein kinase C activator, phorbol 12-myristate 13-acetate (PMA, 0.01 *μ*g/mL final; Biovision, USA) for 1 h. Following PMA stimulation or basal state, cells were incubated with 2 *μ*M SYTOX green to detect extracellular DNA. After 30 min, neutrophils were washed and resuspended in RPMI and the plates were read using the SpectraMAX Gemini fluorescence microplate reader (Molecular Devices, Sunnyvale, CA, USA) at excitation and emission wavelengths of 500 nm and 530 nm, respectively. The data were analyzed using SoftMax Pro Software (Molecular Devices). To measure ROS, isolated neutrophils were incubated at 37°C for 30 min in the presence of 2 *μ*M 2′,7′-dichlorofluorescin diacetate (Molecular Probes), which is used to detect hydrogen peroxide, peroxyl radicals, and peroxynitrite anions. To evaluate NET formation following 1 h PMA stimulation, extracellular fluorescence was measured using a spectrofluorometer at excitation and emission wavelengths of 488 nm and 527 nm, respectively.

### 2.5. Western Blot Analysis

Neutrophil lysates obtained from HV, MHD, and acute infection were tested for histone H3, citrullinated histone H3 (Cit H3) (anti-Histone H3 (citrulline R2 + R8 + R17) antibody, Abcam), and glyceraldehyde 3-phosphate dehydrogenase (GAPDH, internal control) by Western blots. For Western blot analysis, proteins (about 100 *μ*g per lane) were separated by sodium dodecyl sulphate-polyacrylamide gel electrophoresis (SDS-PAGE) on 15% polyacrylamide gels and transferred onto nitrocellulose membranes (Bio-Rad Laboratories, Hercules, CA). The membranes were blocked in 0.05% PBS-Tween (PBST) containing 5% milk (Bio-Rad Laboratories, Hercules, CA) and then incubated with the primary antibody at 4°C overnight.

### 2.6. Statistical Analysis

As an exploratory study, the sample size of the study was planned based on an expected mean difference between MHD and 2 control populations (negative and positive) of 20% of NET formation. Using nonparametric sample size estimation, the sample size calculation for the group sequential design resulted in a sample size of 62 patients in whom control groups were equally divided into 30 patients to achieve a power of at least 80%. Statistical analyses were performed using SPSS version 25.0 software (SPSS Inc., IL, USA). All data are expressed as the mean ± standard deviation (SD) or medians and ranges. Differences between groups were analyzed by the independent *t*-test for continuous parameters. A repeated measures analysis of variance (ANOVA) linear model was used to compare laboratory parameters among the 3 groups, healthy control, MHD patients, and those with acute infection. Correlations between clinical and biochemical factors and baseline NET fluorescence were evaluated using Pearson's correlation or Spearman's rank correlation. With multiple regression analysis, the influences of prevalent CAD and inflammation on baseline NET formation were assessed. Statistical significance was accepted when *p* < 0.05.

## 3. Results

All data are summarized in Table 1. MHD patients had significantly elevated concentrations of serum urea, creatinine, and hs-CRP levels, compared with HV patients. In addition, MHD patients also had significantly higher WBC and circulating neutrophil counts in peripheral blood, compared with HV individuals. As expected, patients with acute infection had the highest levels of WBC, neutrophils, uric acid, and hs-CRP of the three groups.

### 3.1. Basal Activities and Phenotypes of Neutrophils in Dialysis Patients

**Figure 1:**
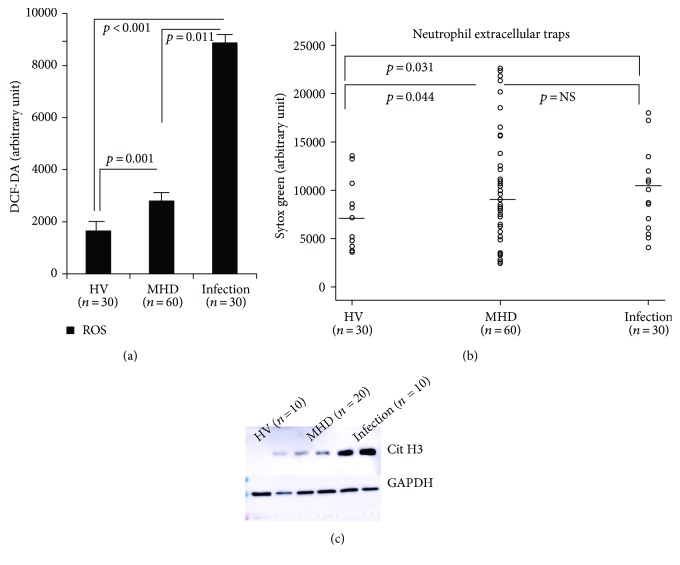
Basal activity of neutrophils of the HV, MHD, and acute infection groups. Compared with HV patients, neutrophils extracted from MHD individuals produced significantly higher levels of ROS (a), basal NET formation (b), and hypercitrullination (c) compared with those from the HV group.

Figure 1(a) shows basal ROS production in neutrophils. Compared with HV patients, neutrophils extracted from MHD individuals produced significantly higher levels of ROS, indicating that they were activated spontaneously. ROS production in MHD patients, however, was lower than that in neutrophils extracted from patients with acute infections (Figure 1(a)). Basal NET formation was also significantly higher in the MHD group, compared with the HV group. The median levels of fluorescence were 5187.3, 7767.6, and 9784.2 in the HV, MHD, and acute infection groups, respectively (Figure 1(b); HV versus MHD, *p* = 0.04; HV versus infection *p* = 0.03). Also, as shown in Figure 1(c), an increase in Cit H3 was detected in the MHD group compared to that in the HV group.

**Figure 2:**
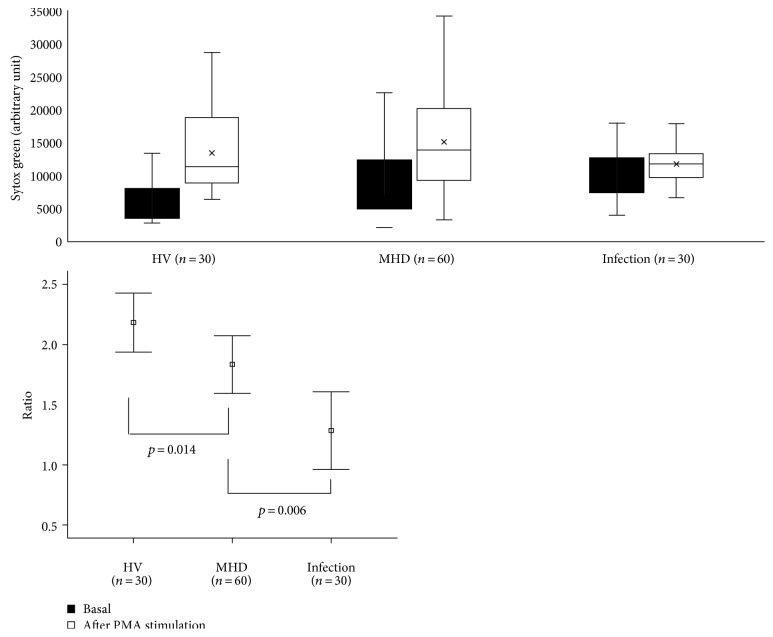
Response of neutrophils to stimulus. NET formation was increased 2.31-fold upon PMA treatment in HV. NET formation among the MHD and acute infection groups, however, was only stimulated 1.89- and 1.28-fold, compared with that observed in the HV group, supporting the spontaneous activated state of neutrophils.

Also, as shown in Figure 2, NET formation was increased 2.31-fold upon PMA treatment in HV. NET formation among the MHD and acute infection groups, however, was only stimulated 1.89- and 1.28-fold, compared with that observed in the HV group (*p* = 0.014 and *p* = 0.006, resp.). These data are not surprising given that neutrophils from patients with acute infections are constitutively activated, and therefore, additional activation may be limited. Similarly, neutrophils from MHD patients are spontaneously activated at baseline and their response to stimulation may also be limited.

**Figure 3:**
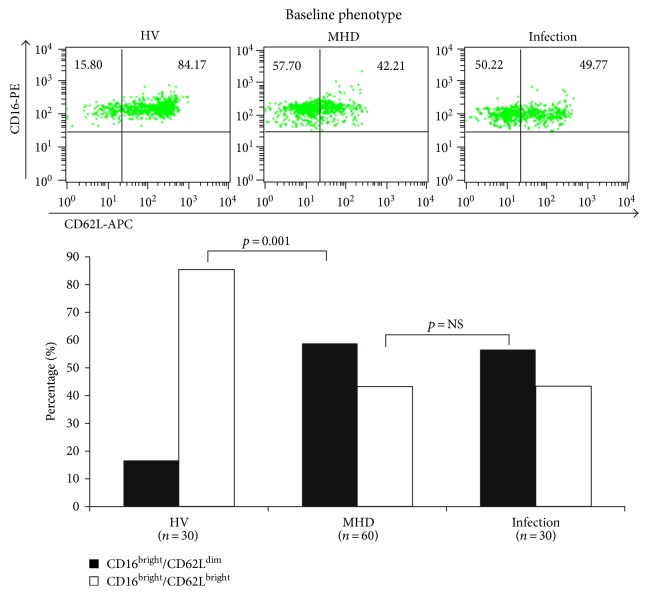
Changes in phenotypes of neutrophils in maintenance hemodialysis patients. Neutrophils from HV patients were normal CD16^bright^/CD62L^bright^ cells; however, neutrophils from MHD patients were CD16^bright^/CD62L^dim^, similar to those from patients with acute infections.

**Figure 4:**
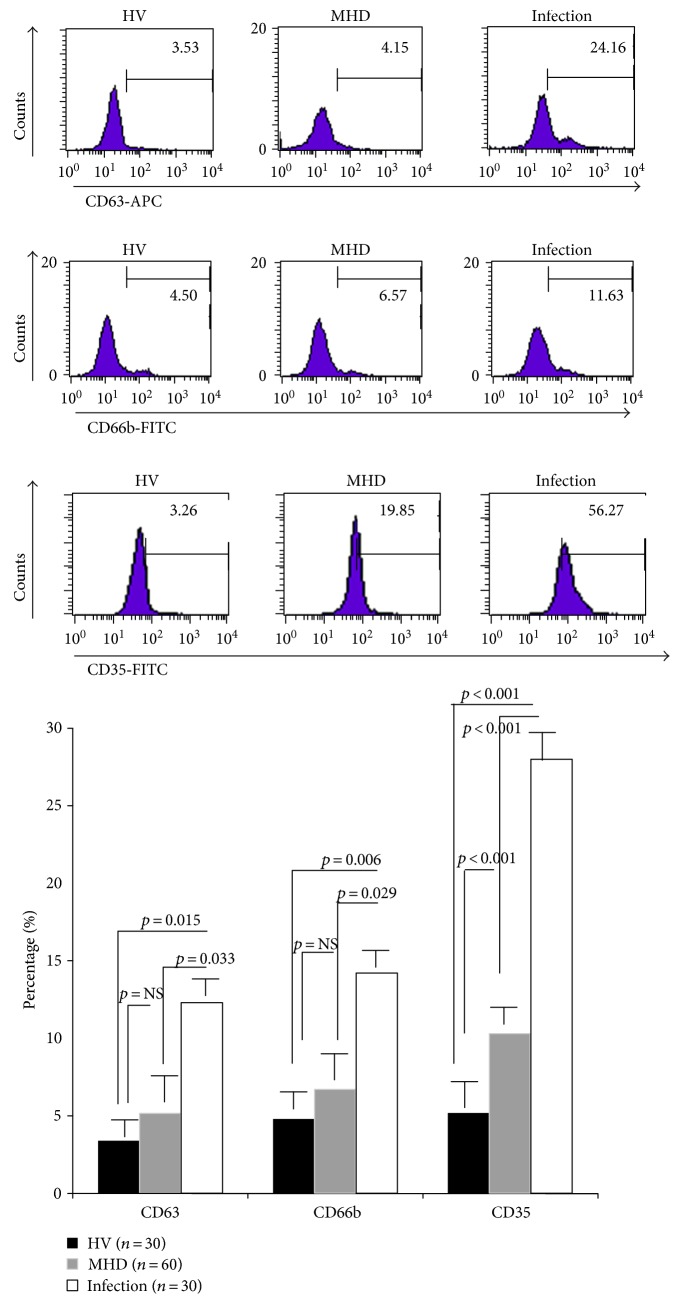
Degranulation of neutrophils at baseline. Expression of CD35 was significantly increased on the surface of neutrophils from MHD patients, compared with HV patients, indicating spontaneous degranulation. All three markers were significantly higher in the acute infection group than in the HV and MHD groups.

And there were significant differences in neutrophil phenotypes among MHD patients. As shown in Figure 3, neutrophils from HV patients were normal CD16^bright^/CD62L^bright^ cells; however, neutrophils from MHD patients were CD16^bright^/CD62L^dim^, similar to those from patients with acute infections. Expression of CD35 was also significantly increased on the surface of neutrophils from MHD patients, compared with HV patients, indicating spontaneous degranulation. The expression of CD63 and CD66b was not significantly different between MHD and HV individuals; however, all three markers were significantly higher in the acute infection group than in the HV and MHD groups (Figure 4).

### 3.2. Correlation with Clinical Characteristics

Among the patients undergoing MHD, mean age was 64.0 ± 13.1 years and 35 patients (58.3%) were male. The causes of ESRD were diabetic nephropathy (32 patients), hypertensive nephrosclerosis (14 patients), chronic glomerulonephritis (7 patients), and others (7 patients). The median length of dialysis was 26.6 months, and 19 (31.7%) had a previous history of CAD. Since variability in baseline NET formation was observed in the MHD group, we tried to find clinical and biochemical parameters associated with increased NET formation at baseline. Patients were divided into 2 groups based on median NET formation (7767.6). As shown in Table 2, increased NET levels were only associated with the previous history of CAD, but not with age, BMI, diabetes, or dialysis duration. Also, blood urea nitrogen and creatinine levels were not associated with NET levels. The median NET formation between MHD patients with and without CAD was 12222.0 (IQR, 7666.0, 18526.5) and 6463.8(IQR, 3812.1, 10200.1), respectively. Among the biochemical parameters, whole blood WBC (*p* = 0.02) and neutrophil (*p* < 0.001) counts were significantly elevated in patients with increased NET formation, as were N/L ratios (*p* = 0.001) and hs-CRP (*p* = 0.01) levels.

Baseline NET formation was positively correlated with CAD, peripheral neutrophil count, and inflammatory markers such as N/L ratio and hs-CRP levels (Table 3). Moreover, multivariate analyses identified the prevalence of CAD and neutrophil counts as independent predictors of baseline NET formation (*β* = 0.323, *p* = 0.01 and *β* = 0.369, *p* = 0.006, resp.).

## 4. Discussion

This study found that basal NET formation was significantly increased in MHD patients and was associated with peripheral blood neutrophil counts, inflammatory markers, and a prevalent CAD. Decreased NET formation in response to external stimuli was also observed and may explain why MHD patients are more susceptible to infection. Neutrophil dysfunction and increased NET formation may contribute to the pathogenesis and risk of cardiovascular complications in MHD patients.

Macrophages have generally been considered the major immune cells involved in the pathogenesis of atherosclerosis; however, the roles of polymorphonuclear neutrophils are becoming increasingly recognized [29], because the first cells to respond to tissue damage are neutrophils. Neutrophils primarily orchestrate the early stages of atherosclerosis by mechanisms involving the release of alarmins, thereby promotes arterial recruitment of classical monocytes and macrophages [29, 30]. Indeed, NETs as well as neutrophils were identified in luminal location in murine and human atherosclerotic lesions [31]. Recently, Warnatsch et al. reported that cholesterol crystals triggered neutrophils to release NETs and these NETs primed macrophages for cytokine release, activating T helper cells that amplify immune cell recruitment in atherosclerotic plaque [32]. That study also identified NETs adjacent to cholesterol crystals in atherosclerotic lesions in apolipoprotein E-deficient mice, suggesting that NETs could act as an endogenous toxin triggering vascular inflammation.

In fact, uremia-associated immune cell dysfunction has been established as a risk factor for viral-associated cancers, infections, and a decreased response to vaccines in patients with renal dysfunction [33–35]. The increased risk of atherosclerosis and vascular complications is also likely related to uremia-associated immune dysfunction and inflammation. Neutrophils are spontaneously activated with abundant uremic toxins and oxidative stress. We hypothesized that such primed neutrophils will release more NETs and aggravate vascular inflammation and atherosclerotic complications, based on the neutrophil-macrophage interactions in atherosclerosis [30], which is exceedingly prevalent in MHD patients. Although the dysfunction of neutrophils has been reported in multiple studies, no clinical investigations have been performed to assess the direct relationship between NET formation and clinical outcome. To our knowledge, this is the first study investigating the potential role of NET formation on inflammation and vascular complications in MHD patients.

This study confirmed the basal activation state of neutrophils in MHD patients. Baseline NET formation and ROS production were significantly elevated in those patients, compared with HV individuals. Nevertheless, the response to external stimuli and the degree of NET formation in response to PMA were significantly decreased in MHD patients. Considering the importance of NET formation on the bactericidal activity of neutrophils, the dysfunction of neutrophils may underlie the higher infection rates among MHD patients.

Also, the phenotype of neutrophils was changed in MHD patients; neutrophils were primarily CD16^bright^/CD62L^dim^, which produce significantly higher levels of ROS and higher levels of NET formation. Such phenotypic changes are consistent with previous studies characterizing neutrophil dysfunction in cerebrovascular inflammation [36] and uremia [37]. These phenotypical changes as well as the spontaneous priming, but reduced bactericidal activities of neutrophils, may be due to the imbalance between apoptosis and necrosis [13]. Some uremic toxins delay apoptosis, causing neutrophils to undergo necrosis, which is associated with the release of numerous proinflammatory molecules [38]. In support of this, significant correlation was observed among NET formation, N/L ratio, and the expression of inflammatory markers, including hs-CRP in our study.

And one more interesting finding in our study is that patients with a previous history of CAD had significantly higher levels of basal NET formation than did patients with no history of CAD. Increased NET formation and the phenotypic changes observed in blood samples represent the in vivo inflammatory conditions associated with coronary atherosclerosis. Although a causal relationship between the prevalence of CAD and neutrophil dysfunction with increased NET formation was not identified in this study, an important link between these factors and cardiovascular disease is nonetheless likely. In addition, the numbers of circulating neutrophils in peripheral blood was also correlated with baseline NET formation in this study. The number of circulating neutrophils was significantly higher in patients with MHD compared with HV. Thus, an increase in circulating neutrophil count may be related to neutrophil activation and inflammation, indicative of underlying CAD or vascular atherosclerosis in HD patients.

Our study has several limitations. First, CAD was defined as significant coronary stenosis with coronary intervention (percutaneous coronary intervention or coronary bypass graft surgery). However, the prevalence of silent ischemia is frequent in ESRD patients. Therefore, the prevalence of CAD may be underestimated in this study. And, although MHD patients with an acute or recent infection were excluded in study design, the possibility of having chronic infection such as gingivitis or asymptomatic urinary tract infection cannot be completely excluded. These chronic infections may influence the neutrophil activity. Lastly, due to the small number of included study, this is an explorative study.

## 5. Conclusions

The study provides direct evidence of spontaneous neutrophil activation with increased NET formation in MHD patients and their close relationship with in vivo inflammatory conditions and prevalent CAD. Additionally, the number of circulating neutrophils may also be a reflection of the overall neutrophil activation and could be a sign of atherosclerotic vascular complications in ESRD.

## Conflicts of Interest

There is no financial conflict of interest in this study.

## Figures and Tables

**Table 1:** Biochemical parameters in HV, MHD, and infection groups.

Variables	HV (*n* = 30)	MHD (*n* = 60)	Infection (*n* = 30)
WBC count (/*μ*L)	4813 ± 1006	6180 ± 1807^∗^	18815 ± 3991^∗∗^
Neutrophil count (/*μ*L)	3115 ± 1101	4374 ± 1694^∗^	17750 ± 6540^∗∗^
Hemoglobin (g/dL)	13.8 ± 0.3	10.3 ± 0.9^∗^	8.6 ± 2.1^∗∗^
Urea nitrogen (mg/dL)	16.0 ± 0.9	52.6 ± 20.4^∗^	38.2 ± 13.6^∗∗^
Creatinine (mg/dL)	0.8 ± 0.2	8.2 ± 3.4^∗^	1.6 ± 0.5^∗∗^
Albumin (g/dL)	4.1 ± 0.1	3.6 ± 0.5^∗^	3.1 ± 1.1^∗∗^
Uric acid (mg/dL)	4.6 ± 1.8	6.9 ± 1.6^∗^	7.8 ± 1.5^∗^
Cholesterol (mg/dL)	170.1 ± 7.5	139.0 ± 28.7^∗^	110.8 ± 21.1^∗∗^
hs-CRP (mg/L)^†^	−0.21 ± 0.6	0.40 ± 1.13^∗^	16.1 ± 10.6^∗∗^
Intact PTH (pg/mL)	—	181.5 ± 149.2	—
*β*2-microglobulin	—	22.9 ± 8.4	—
spKt/V	—	1.60 ± 0.28	—
nPCR (g/kg/day)	—	1.20 ± 0.13	—

^∗^ *p* < 0.001 compared to HV, ^∗∗^*p* < 0.001 compared to HV and MHD.

^†^Log transformed.

**Table 2:** Comparisons of baseline characteristics according to median level of baseline NET in MHD patients.

		Baseline NET (median 7767.6)		
Variables	Total	<median level	≥median level	*p*
Age (years)	64.0 ± 13.0	62.4 ± 13.2	65.5 ± 13.0	0.35
Gender, male, *n* (%)	35 (58.3)	18 (62.1)	17 (54.8)	0.38
BMI	22.9 ± 4.4	22.6 ± 4.2	23.2 ± 4.6	0.61
Diabetes	35 (58.3)	17 (58.6)	18 (58.1)	0.59
Duration of dialysis (months)	49.4 ± 26.6	54.1 ± 25.2	41.9 ± 28.4	0.22
Systolic blood pressure (mmHg)	145.0 ± 19.8	141.9 ± 20.4	148.0 ± 19.4	0.24
Diastolic blood pressure (mmHg)	81.3 ± 10.7	79.0 ± 8.6	83.4 ± 12.2	0.11
Previous CAD, *n* (%)	19 (31.7)	4 (13.8)	15 (48.4)	**0.004**
Previous cerebrovascular accident, *n* (%)	9 (15.0)	2 (6.9)	7 (22.6)	0.08
WBC count (/*μ*L)	6180 ± 1807	5622 ± 1896	6700 ± 1578	**0.02**
Neutrophil count (/*μ*L)	4374 ± 1694	3482 ± 994	5302 ± 1777	<**0.001**
Lymphocyte count (/*μ*L)	1271 ± 479	1370 ± 479	1168 ± 460	0.13
N/L ratio	4.2 ± 3.0	2.7 ± 0.9	5.8 ± 4.4	**0.001**
Hemoglobin (g/dL)	10.3 ± 0.9	10.5 ± 0.9	10.2 ± 0.9	0.14
Urea nitrogen (mg/dL)	52.6 ± 20.4	55.1 ± 22.3	50.3 ± 18.5	0.36
Creatinine (mg/dL)	8.2 ± 3.4	8.9 ± 3.5	7.6 ± 3.1	0.17
Albumin (g/dL)	3.6 ± 0.5	3.6 ± 0.6	3.6 ± 0.5	0.76
Uric acid (mg/dL)	6.9 ± 1.6	6.9 ± 1.9	6.8 ± 1.4	0.86
Cholesterol (mg/dL)	139.0 ± 28.7	145.9 ± 29.2	132.6 ± 27.7	0.07
hs-CRP (mg/L)^∗^	0.40 ± 1.13	0.03 ± 0.94	0.75 ± 1.20	**0.01**
Intact PTH (pg/mL)	181.5 ± 149.2	201.0 ± 158.8	163.1 ± 139.6	0.33
*β*2-microglobulin	22.9 ± 8.4	20.6 ± 5.8	25.7 ± 10.2	**0.06**
spKt/V	1.60 ± 0.28	1.57 ± 0.23	1.64 ± 0.33	0.42
*Medications, %*				
Aspirin	56.8%	59.1%	55.5%	0.78
Lipid-lowering agent	35.0%	33.3%	37.1%	0.44

All data are expressed as mean ± SD except for those with ∗ which are expressed as median with range.

BDI-II: Beck depression inventory II; BMI: body mass index; ESRD: end-stage renal disease; intact PTH: intact parathyroid hormone; TSH: thyroid-stimulating hormone; hs-CRP: high-sensitivity C-reactive protein; spKt/V: single pool Kt/V; nPCR: normalized protein catabolism rate.

**Table 3:** Correlation analysis of baseline NET levels with clinical parameters.

	NET	
	Correlation coefficient	*p*
Age	0.101	0.44
Gender	0.134	0.31
Dialysis duration	0.009	0.94
Diabetes	0.095	0.47
CAD	**0.470**	<**0.001**
Cerebrovascular accident	0.139	0.29
WBC count (/*μ*L)	**0.246**	**0.05**
Neutrophil count (/*μ*L)	**0.486**	<**0.001**
Lymphocyte count (/*μ*L)	−0.201	0.17
N/L ratio	**0.364**	**0.009**
Hemoglobin (g/dL)	−0.156	0.23
Cholesterol	−0.219	0.09
hs-CRP (mg/L)^∗^	**0.284**	**0.02**
*β*2-microglobulin	0.234	0.16
spKt/V	0.063	0.71

BDI-II: Beck depression inventory II; hs-CRP: high-sensitivity C-reactive protein; BMI: body mass index.

^*∗*^A log-transformed value.
